# LncRNAs and CircRNAs in Endoplasmic Reticulum Stress: A Promising Target for Cardiovascular Disease?

**DOI:** 10.3390/ijms24129888

**Published:** 2023-06-08

**Authors:** Francisco José Martinez-Amaro, Carlos Garcia-Padilla, Diego Franco, Houria Daimi

**Affiliations:** 1 Department of Experimental Biology, University of Jaen, 23071 Jaen, Spain; fmamaro@ujaen.es (F.J.M.-A.); cgpadill@ujaen.es (C.G.-P.); dfranco@ujaen.es (D.F.); 2 Department of Human Anatomy and Embryology, Faculty of Medicine, Institute of Molecular Pathology Biomarkers, University of Extremadura, 06006 Badajoz, Spain; 3 Medina Foundation, 18016 Granada, Spain; 4 Laboratory of Human Genome and Multifactorial Diseases (LR12ES07), Faculty of Pharmacy, University of Monastir, Monastir 5000, Tunisia; 5 Department of Biology, Faculty of Sciences, University of Gabes, Gabes 6072, Tunisia

**Keywords:** non-coding RNAs, ER stress, UPR, ERAD, apoptosis, autophagy, cardiovascular diseases

## Abstract

The endoplasmic reticulum (ER) is a principal subcellular organelle responsible for protein quality control in the secretory pathway, preventing protein misfolding and aggregation. Failure of protein quality control in the ER triggers several molecular mechanisms such as ER-associated degradation (ERAD), the unfolded protein response (UPR) or reticulophagy, which are activated upon ER stress (ERS) to re-establish protein homeostasis by transcriptionally and translationally regulated complex signalling pathways. However, maintenance over time of ERS leads to apoptosis if such stress cannot be alleviated. The presence of abnormal protein aggregates results in loss of cardiomyocyte protein homeostasis, which in turn results in several cardiovascular diseases such as dilated cardiomyopathy (DCM) or myocardial infarction (MI). The influence of a non-coding genome in the maintenance of proper cardiomyocyte homeostasis has been widely proven. To date, the impact of microRNAs in molecular mechanisms orchestrating ER stress response has been widely described. However, the role of long noncoding RNAs (lncRNAs) and circular RNAs (circRNAs) is just beginning to be addressed given the potential role of these RNA classes as therapeutic molecules. Here, we provide a current state-of-the-art review of the roles of distinct lncRNAs and circRNAs in the modulation of ERS and UPR and their impact in cardiovascular diseases.

## 1. Introduction

The endoplasmic reticulum (ER) is the largest, multifunctional, membrane-like, cellular organelle, composed of smooth and rough ER and forms an interconnected network of space [1]. ER exerts a pivotal role in three physiological cellular processes: (1) modulation of correct protein secretion, folding and translocation from ER lumen, (2) regulation of intracellular Ca^2+^ uptake, storage and signalling and (3) production of several membrane cellular lipids such as cholesterol, ceramides and/or glycerophospholipids [2,3].

A significant percentage of intracellular proteins are synthesised in ER lumen, wherein its oxidative environment facilitates the formation of disulphide bonds on proteins by different chaperones, foldases and cofactors. Generating disulphide bonds leads to proper secretory and transmembrane protein folding [4,5]. Alteration of ER protein folding capacity may cause an increased proportion of unfolded and misfolded proteins in ER lumen which triggers loss of ER homeostasis and proteostasis and generates a detrimental cellular environment [6,7,8]. Several molecular and biophysical mechanisms are triggered to reverse and restore ER homeostasis such as (1) ER-associated degradation (ERAD), which triggers the misfolded protein degradation from ER lumen; (2) Unfolded protein response (UPR) involving the restoration of ER proteostasis by activation of three transduction signalling –IRE1, ATF6 and PERK branch-; and (3) Reticulophagy, the process of ER remodelling by autophagy of membranes and associated proteins (see reviews [9,10,11,12,13,14]). Pathophysiological factors occurring in cardiovascular diseases (CVDs) such as metabolic derangement, hypoxia, hypertrophy or inflammation require an increased protein expression, thus enhancing the disruption of the cellular proteostasis [15,16,17,18,19,20,21]. As a consequence of the increased requirement of protein synthesis, ER homeostasis is ruptured and different subpopulations of cardiac cells suffer an unfolded and misfolded protein accumulation, which in turn, induces ER stress [22,23,24,25]. Accumulation of deleterious proteins triggers ER stress signalling which exerts a bivalent role both beneficial and/or harmful in cardiovascular function [26,27,28,29,30,31,32,33]. Furthermore, ER homeostasis is closely associated with normal cardiovascular function, and ER stress is considered a cause and a consequence of an extensive variety of CVDs such as ischaemic heart disease, hypertension, heart failure and dilated cardiomyopathy [34,35,36,37]. Here, we address an exhaustive current state-of-the-art of the impact of ncRNA in ERS-related cardiovascular diseases focusing on the role of distinct lncRNAs and circRNAs, described to date, on the modulation of UPR signal and their function in cardiovascular disease progression.

## 2. ERS and UPR Signalling

**Figure 1:**
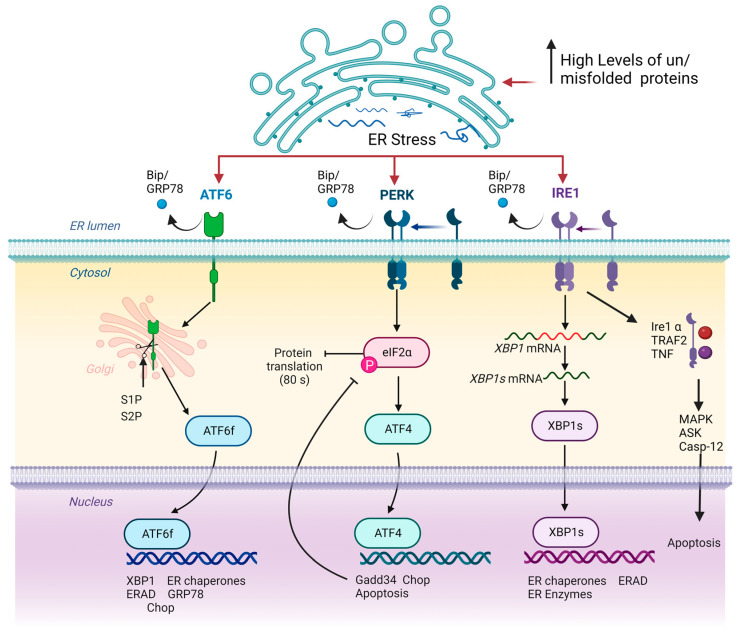
Schematic representation of UPR signalling pathways. Note that in homeostatic conditions, IRE1, ATF6 and PERK remain attached to ER membrane, exerting a sensorial cellular function. Loss of ER homeostasis by increased concentration of misfolded or unfolded proteins triggers the delivery of IRE1, ATF6 and PERK proteins to ER membrane by Bip/GRP78 factor. Subsequently, ATF6 is modified, acquiring transcriptional activity, while IRE1 and PERK activate ATF4 and XBP1s, respectively, which in turn exerts a transcriptional function. Inside the nucleus, ATF6, ATF4 and XBP1s initiate the expression of several genes that aim to restore cellular proteostasis. Arrows and bar-headed lines represent activation and inhibition effects respectively.

Since ER is crucial for the correct functioning of the cell, there are ER stress response mechanisms that control the degradation of the unfolded or misfolded proteins aiming to maintain ER homeostasis. The core mechanism of control is the activation of unfolded protein response (UPR). The central function of UPR is the inhibition of protein synthesis and the increase in the folding capacity of the ER. UPR may be activated by three different signal transduction pathways, initiated by three proteins located in ER membrane: inositol requiring protein 1 (IRE1), protein kinase RNA-like ER kinase (PERK) and activating transcription factor 6 (ATF6). In basal conditions, these molecules are bound to a chaperone named Bip (or GRP78) and remain attached to the ER membrane. However, when misfolded or unfolded proteins are accumulated, they dissociate and trigger three different signalling pathways induced by IRE1, PERK and ATF6 to resolve ER stress (Figure 1) [38,39].

IRE1 is the most conserved factor across evolution involved in the UPR pathway. IRE1 possess an endoribonuclease activity domain responsible for its molecular function and is represented by two isoforms, IRE1α and IRE1β. IRE1 is activated by auto-phosphorylation and homodimerisation under the loss of ER homeostasis. Activated IRE1 is delivered to ER membrane and recognises a consensus region in the X-box binding protein 1 (XBP1) mRNA, inducing alternative splicing by cleavage of a 26-nucleotide intron. Such a cleavage results in a functional active protein XBP1 named, XBP1s. XBP1s exerts as a transcription factor triggering expression of several UPR target genes such as ERAD components, ER chaperones, ER-translocation and folding enzymes further reducing ER stress levels. However, maintenance of IRE1α results in increased apoptosis. IRE1α interacts with tumour necrosis factor receptor-associated factor 2 (TRAF2) and adaptor protein tumour necrosis factor (TNF) to form a complex [40]. This complex recruits mitogen-activated protein kinase (MAPK), apoptosis signal-regulating kinase (ASK), and caspase-12 in order to trigger apoptosis [41,42].

Like IRE1, activation of PERK occurs by autophosphorylation of its kinase domain. Activated PERK modulates phosphorylation of eukaryotic translation initiation factor 2 alpha (Eif2α), which in turn, inhibits 80S ribosome assembly and thus protein synthesis, inducing a reduction in the ERS. Furthermore, Eif2α enhances the translation of activating transcription factor 4 (Atf4) mRNA. Atf4 induces the transcription of growth arrest and DNA damage-inducible protein 34 (Gadd34) and c/EBP homologous protein (CHOP) resulting in the activation of several proapoptotic signalling. CHOP induces apoptosis by the induction of several caspases and proapoptotic factors. Curiously, Gadd34 regulates the dephosphorylation of Eif2α when ER stress is solved, and it restores the normal protein translation. Dephosphorylation of Eif2α is required to conduct prosurvival signalling.

Activation transcription factor 6 (ATF6) is an ER transmembrane protein belonging to the leucine zipper transcription factor family. ATF6 acts as a core modulator of autophagy and apoptosis in response to increased ER stress [43]. When the ER is stressed, ATF6 is delivered from ER membrane by Bip and transported to the Golgi apparatus, where it is cleaved by two different proteases, site-1 protease (S1P) and site-2 protease (S2P), generating a 50 kDa amino-terminal cytoplasmic fragment and acquiring a transcriptional activation function (ATF6f). ATF6f is capable to enter the nucleus and trigger the expression of ERAD components, GRP78 and XBP1. Furthermore, ATF6 may bind to the endoplasmic reticulum response element (ERSE) and thereby activating CHOP and inducing cell apoptosis in several pathologies [44].

Early activation of UPR—named adaptive UPR—exerts a protective role against several injuries promoting cell survival and improving cellular function. Furthermore, UPR is required for different cellular processes such as differentiation and proliferation, pinpointing an important role in appropriate development and cellular physiology [45,46,47,48]. For example, activation of three branches of UPR- IRE1, PERK and ATF6- is necessary for the expression of several myogenic genes such as *Mef2c* or *MyoD*, the correct formation of myotubes and therefore proper embryonic myogenesis [49,50,51,52]. In addition, the regeneration of skeletal muscle by activation of satellite cells requires the expression of PERK signalling and downstream genes suggesting a crucial role in the regenerative process [53]. Beneficial and physiological effects observed by adaptive UPR are closely related to the maintenance of calcium homeostasis, mitochondrial function and the regulation of homeostatic levels of free radicals in the cell cytoplasm [54,55,56]. However, prolonged stimulation of the UPR signalling pathway—known as maladaptive UPR—has a deleterious effect on cellular homeostasis increasing cellular apoptosis, ROS generation and impaired cell function thus displaying a detrimental role in several pathologies [57,58,59,60,61].

Complementary to the previously described mechanisms, there are also alternative processes that resolve ER stress and support UPR protective function such as ER-associated degradation (ERAD). ERAD is an evolutionarily and anciently conserved mechanism which modulates the degradation of misfolded or unfolded proteins from ER resulting in a subsequent reduction in the ERS. In this process, the misfolded or unfolded proteins accumulated in the ER are translocated to the cytosol where they are ubiquitinated and degraded by the proteasome. 

ERAD substrates are recognised by different ligases and chaperones depending on whether the misfolded or unfolded domain of the protein is located in the ER lumen, within the ER membrane, or on the cytosolic side of the membrane (ERAD-L, ERAD-M and ERAD-C, respectively). ERAD-L and ERAD-M are driven by Hrd1—RING-finger ligase—a core ubiquitin ligase that forms a protein complex with other ligases such as Hrd3, Usa1 or Der1. Whereas ERAD-C substrates are targeted by Doa10p ligase.

Hrd1 protein is formed by six transmembrane domains and a cytoplasmic tail in which a catalytic RING finger is necessary for E3 ligase activity. Curiously, the transmembrane regions of Hrd1 may form a retrotranslocation channel to export ER proteins. The RING finger domain is located in the cytosol to serve at least two distinct purposes. First, Hrd1-dependent autoubiquitination of the RING finger domain gates its own channel function. This finding raises the possibility that deubiquitinases might counter the ubiquitination reaction and control the retrotranslocation event as well. Whether autoubiquitination is a general feature that regulates the channel activity of other E3 ubiquitin ligases dedicated to ERAD is unclear at this point. Second, Hrd1 catalyses ubiquitination of the misfolded substrates once exposed to the cytosol, which in turn are tagged for proteasomal degradation. Recently another E3 ligase gp78 has been described acting downstream, or in parallel, to the Hrd1-ligases complex, enhancing the solubility of the retrotranslocated protein substrates by proper proteasomal degradation.

Another important mechanism to resolve ER stress is reticulophagy, a type of macro-autophagy leading to the removal of excess unfolded and misfolded proteins from ER lumen. This process consists of the creation of autophagosomes specifically from ER membranes in order to remove excess deleterious proteins of ER. Several molecular mechanisms of reticulophagy have been described [62,63,64]. Increased unfolded and misfolded proteins trigger auto-ubiquitination of the E3 ubiquitin-protein ligase tripartite motif-containing protein 13 (TRIM13) which recruits autophagy adaptor sequestosome 1 (p62). The oligomerisation of both proteins is dependent on the binding of N-Degron to the ZZ domain from p62. TRIM13-p62 protein complex oligomerisation is required to recruit LC3B and other chaperones involved in reticulophagy. LC3B induces specific reticulophagy of ER portions enriched in folding elements and chaperones involving lysosome-associated membrane glycoprotein 1 (LAMP1), RAB7 (in ER- engulfing endolysosomes), charged multivesicular body protein 4B (CHMP4B) and vacuolar protein sorting-associated protein 4A (VPS4A) [45,65,66,67,68]. Furthermore, the PERK-EIF2A pathway is responsible for the activation of the ATG12-ATG16-ATG5 complex which in turn establishes a signature mark into autophagy membranes by converting LC3-I into LC3-II [69]. Like ERAD or UPRs, excessive removal of ER membranes could be translated into the disruption of autophagy and increased apoptosis [70,71].

## 3. Role of ERS and UPR in Cardiovascular Diseases

ERS and subsequent activation of UPR exhibit both beneficial and deleterious effects in cardiovascular diseases, being thus considered both as a cause and consequence of them. Cardiac pathologies increase the demand and requirements of the ER function since an enhanced proportion of misfolded proteins triggers in many cases the loss of homeostasis of this organelle. Furthermore, ERS exerts a pivotal role in the modulation of both Ca^2+^ homeostasis and mitochondrial function in cardiomyocytes. Prola et al. (2019) have demonstrated that Tunicamycin (TM) treated cardiomyocytes display several changes in their cytoplasm ultrastructure, such as enlarged cytosol, decreased mitochondrial number, increased proportion of mitochondria-associated-membrane (MAM) fraction and expansion and dislocation of the ER near to nucleus and thus away from the sarcomeres. Accordingly, ERS reduced the mitochondrial number and function by downregulating several proteins involved in mitochondrial biogenesis such as PGC1a, TFAM, NRF1 or CS and thus is involved in the reduction of the mitochondrial capability to produce ATP [72]. Initially, adaptive UPR activation is capable of restoring ER and mitochondrial function and thus sustaining cardiac homeostasis. Curiously, the effects of molecular signalling pathways triggered by ERS are different within distinct cardiovascular injuries such as atherosclerosis, myocardial infarction, heart failure, cardiac hypertrophy or ischaemia and reperfusion (I/R) injury among others. For example, in heart failure or hypertrophy cardiac response caused by cardiac pressure overload, the PERK signalling pathway increases autophagy while it reduces ROS levels and apoptosis ratio by upregulation of EIF2A and ATF4, which in turn restores protein-folding capacity [31,73]. A sustained upregulation of the axis EIF2A-ATF4 will produce an increase in the cardiomyocyte apoptosis triggered by CHOP and these processes can influence the progression of cardiac diseases. In addition, PERK restores Ca^2+^ intracellular concentration by modulating Serca2a and Calreticulin, demonstrating its requirement for a proper ER-dependent ion homeostasis [74]. Unlike PERK, ATF6 is involved in the progression of cardiac hypertrophy and heart failure response thus exerting a harmful role. However, a protective role of ATF6 has been described in I/R injury suggesting a dependent and complex function of UPR based on the type of cardiac injury.

**Figure 2:**
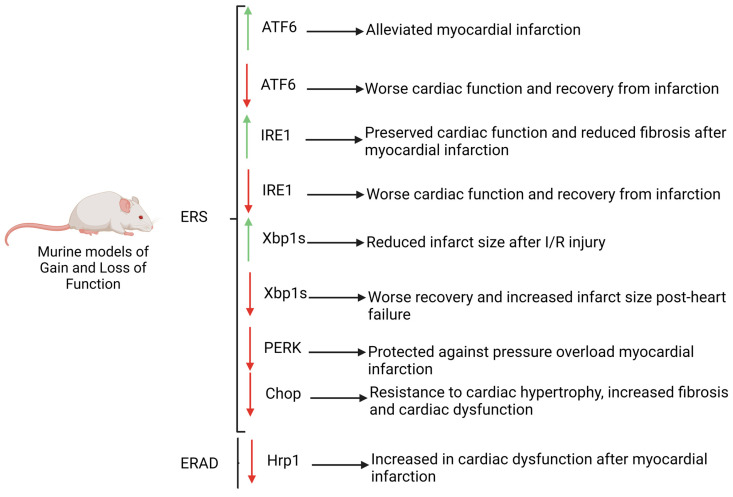
Murine models of gain and loss of function of key genes involved in UPR and ERAD pathways. Note that the deficiency of ATF6, IRE1, CHOP and HRP1 promotes the progression of CVDs, whereas low levels of PERK exert a protective role. Red arrows: downregulation, green arrows: upregulation.

Effects of UPRs’ downstream pathways have been elucidated using several murine models, which have highlighted the importance of ER stress and dependent molecular mechanisms in cardiac homeostasis and pathology (Figure 2). Curiously, ATF6 deficient mice display a worse cardiac function and recovery from infarction after (I/R) injury and increased damage with respect to controls [26]. Furthermore, ATF6 gain-of-function mice exhibits an alleviated myocardial infarction after I/R injury demonstrating that ATF6 is required to protect the heart from damage and injury caused by myocardial infarction [75]. Like ATF6, Xbp1s deficient mice display a worse recovery from heart failure showing an increased infarct size while in vivo overexpression of this gene is translated into reduced infarct size after I/R injury. Similar to that observed in ATF6 and Xbp1s overexpression mouse models, in vivo gain-of-function of Ire1 results in preserved cardiac function and reduced fibrosis after myocardial infarction [27,76]. Unlike IRE1 or ATF6, PERK deficiency has a beneficial phenotype after heart failure displaying protection against pressure overload myocardial infarction suggesting that while ATF6 and IRE1 exert a protective role against heart failure, PERK and its downstream pathways are detrimental [74]. Furthermore, *PERK* is a key gene involved in the transcription activation of CHOP, an essential factor to trigger ERS-associated apoptosis. CHOP-deficient mice are resistant to cardiac hypertrophy, increased fibrosis and cardiac dysfunction pinpointing the importance of apoptosis in deleterious processes related to cardiovascular diseases [77]. Furthermore, loss of function of enzymes related to ERAD signalling have been carried out, reflecting the importance of this mechanism in cardiovascular diseases. For example, Hrp1 deficient mice display an exacerbated cardiac dysfunction after myocardial infarction demonstrating that loss of one mechanism either UPR signalling or ERAD components is enough to impede recovery from cardiac injury [78].

Accordingly, different murine models have proved that the three main pathways involved in ER stress signalling—ATF6, IRE1 and PERK—may play crucial roles in the progression of cardiovascular diseases exerting either protective roles such as in the case of ATF6 or IRE1, or deleterious roles, in the case of PERK. In addition, cardiac dysfunction related to Hrp1 double knockout (dKO) mutant mouse pinpoints the importance of ERAD signalling in cardiac homeostasis.

## 4. Impact of LncRNAs and CircRNAs in ERS and UPR Response on Cardiovascular Diseases

Regulation of ERS and UPR response is the result of crosstalk between several molecular pathways, including therein transcriptional and/or post-transcriptional modulators. Over the last years, several authors have described a pivotal role of non-coding elements in the modulation of UPR signalling pathways, repressing or enhancing it in distinct cardiovascular diseases, particularly microRNAs. Although the role of microRNAs has been widely described [79,80,81,82,83,84], the impact of lncRNAs and circRNAs in ERS regulation and UPR in several cardiovascular diseases is just beginning to be addressed. To date, only seven lncRNAs and one circRNA have been described as pivotal modulators in cardiovascular diseases associated with increased ERS (Table 1). For example, in myocardial infarction (MI), two lncRNAs have been described to exert opposite actions in the progression of this disease namely MEG3—acting as a harmful regulator—and discrimination antagonising non-protein coding RNA (DANCR), which modulates a protective pathway against maladaptive UPR. In addition, an LncRNA—UCA1—and a circRNA—rcDLGAP4—have been described to play important roles in the regulation of the apoptosis induced by blood flow restoration after MI. In the same way, in other cardiovascular pathologies such as atherosclerosis, cardiac hypertrophy, heart failure and dilated or diabetic cardiomyopathy, lncRNAs HypERlnc, NRB2, AC061961.2 and H19 exert different functions in the progression of these diseases. 

Curiously, all of the previously described ncRNAs exert their effect as regulators of UPR response by either activating or repressing ATF6, PERK and IRE1 pathways at different levels but no lncRNAs or circRNAs have been reported related to reticulophagy or ERAD processes.

### 4.1. Atherosclerosis

**Figure 3:**
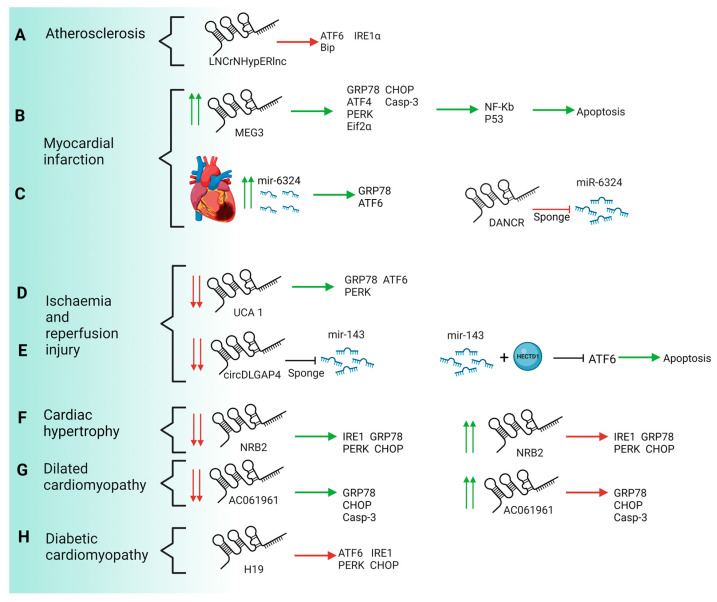
Schematic representation of molecular mechanisms of ncRNAs associated with CVDs. Note that while MEG3 (**B**) (and miR-62324 (**C**)) exerts a harmful role in ERS context, the rest of the lncRNAs currently reported are acting as protective molecules against ERS-mediated apoptosis. HypERlnc, UCA1, NRB2 and AC061961 are downregulated in pathological conditions such as atherosclerosis (**A**), I/R injury (**D**), cardiac hypertrophy (**F**) and dilated cardiomyopathy (**G**), respectively. Particularly, upregulation of NRB2 and AC061961 in vitro results in downregulation of ERS markers such as PERK, IRE1, GRP78 and CHOP. In addition, H19 plays a protective role against diabetic cardiomyopathy by repressing ER stress (**H**). Downregulation of circDLGAP4 leads to reduced ERS through miR-143-HECTD1 mediated inhibition of ATF6 branch (**E**). Red arrows: downregulation, green arrows: upregulation.

**Figure 4:**
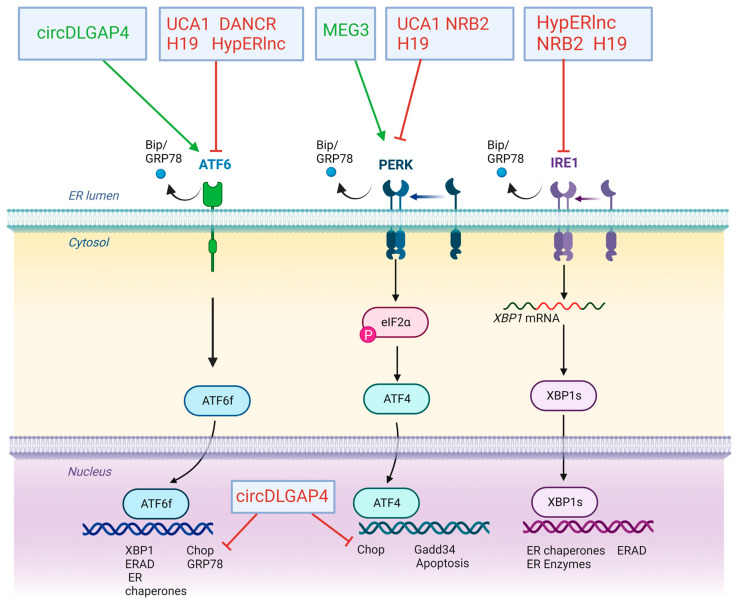
Schematic representation of ERS-associated lncRNAs and circRNAs role modulating UPR pathways in CVDs. Note that these LncRNAS and circRna play an inhibitor or enhancer role over the different molecules involved in the UPR pathway. Particularly, ATF6 pathway is enhanced by CircDLGAP4 and inhibited by UCA1, H19, HyperLnc and DANCR. Perk pathways are activated by MEG3 and inhibited by NRB2, H19 and UCA1. Finally, HyperLinc, H19 and NRB2 are reported to inhibit IRE1.

Atherosclerosis is one of the main causes of cardiovascular diseases all over the world. It may be defined as the accumulation of fibrous materials and/or fatty acid in the deeper layer of the arteries, the intima layer, in addition to endothelial dysfunction and inflammation. This accumulation can produce a structure named atheroma or atheroma plaque whose growth can encroach the arterial lumen and hinder the blood flow [93]. Factors such as hyperlipidaemia, oxidative stress and calcium misbalance can alter ER homeostasis, and trigger ER stress. This state can induce atherosclerosis through different processes such as inflammation and apoptosis, among other factors [79]. The first report on the importance of lncRNAs in cardiovascular disease was provided by Bischoff et al. (2017) describing that HypERlnc, a previously unknown lncRNA annotated as ENSG00000262454, represents a pivotal repressor of UPR by promoting the inhibition of ATF6, IRE1α and Bip transcriptional activation on pericytes exposed to hypoxia. Expression of HypERlnc was significantly downregulated in human cardiac tissue from patients with heart failure (HF). Furthermore, HypERlnc expression was significantly correlated with pericyte markers in human lungs derived from idiopathic pulmonary arterial hypertension patients. In addition, the loss of function of HypERlnc demonstrated that this lncRNA is essential for proper phenotype maintenance, proliferation and survival of pericytes [85]. However, the molecular mechanisms underlying the HypERlnc function have not been described (Figure 3A and Figure 4).

### 4.2. Myocardial Infarction

Myocardial infarction is the main cause of disability or death in the world, may be a cause of instant death or decline of the heart capacity and it is usually preceded by atherosclerosis. Myocardial stroke results in cardiomyocyte cell death due to hypoxia or ischemia caused by an unbalance between the oxygen deposition and requirement in the heart. This ischemia may be caused by an occlusion of the coronary artery with consequent cell death and inflammation [94,95,96].

In 2019, Li et al. analysed the possible relation between lncRNA MEG3 and myocardial infarction pathology. Curiously, expression of MEG3 is increased in both infarcted hearts and hypoxic neonatal mice ventricular myocytes suggesting a possible role in MI. The same report showed that a decrease in MEG3 produces an improvement in cardiac function, a higher fractional shortening, ejection fraction and a lower left ventricular end-systolic and diastolic diameter. Like the MI model, hypoxic neonatal mice ventricular reduction of MEG3 expression alleviates cytotoxicity in the cells and improves cell viability [86].

Mechanistically, knockout of MEG3 reverses apoptosis by repression of several ERS markers such as GRP78, ATF4, PERK, eiF2α, CHOP and caspase 3. Transcriptional activation of genes involved in ERS-associated apoptosis is regulated by several transcription factors such as p53 or NF-Kb. MEG3 is capable of recognising p53, facilitating the binding of this transcription factor to genomic targets, and promoting the transcription of p53-dependent genes such as *NF-Kb*, which in turn enhances the expression of ERS-apoptosis genes. These results suggest that lncRNA MEG3 knockdown exerted cardioprotection by reducing ERS-mediated apoptosis through targeting p53 post-MI [86] (Figure 3B and Figure 4).

Unlike MEG3, DANCR lncRNA exerts a protective role against cardiomyocyte apoptosis in MI. Interestingly, the expression of DANCR is downregulated by tunicamycin (TM) in a concentration-dependent manner, suggesting a possible function in ERS. TM treatment induces ERS-associated apoptosis by increasing expression levels of Bax, cleaved (c)-caspase-3/9, GRP78, IRE1α, Xbp1s, ATF6, ATF4 and Beclin 1. Functional assays demonstrated that TM-treated H9C2 cells display a higher level of apoptosis and lower levels of cell viability, proliferation and autophagy. Curiously, overexpression of DANCR is capable of reversing the effects of TM treatment by reducing the expression of several ERS markers such as GRP78, and Beclin 1, while increasing the expression of apoptotic proteins Bcl-2. Furthermore, DANCR increases p-IRE1α, p-IRE1α/IRE1α and Xbp1s and decreases Xbp1u expression levels, suggesting that DANCR selectively activates the IRE1α pathway in the UPR, promoting autophagy and ERAD, and thus alleviating ERS. Mechanistically, DANCR acts as sponge lncRNA by recognising miR-6324, avoiding thus its binding to mRNA targets. miR-6324 is upregulated in MI and exerts a deleterious role in the progression of this pathology. Additionally, the upregulation of miR-6324 is capable of reversing the protective role of DANCR by increasing cardiomyocyte apoptosis and inducing transcriptional activation of GRP78 or ATF6, thus suggesting an opposite role of ERS-induced TM treatment [63] (Figure 3C and Figure 4).

Related to the progression of MI, an important consequence of the restoration of the blood flow is I/R injury, after a myocardial infarction it is necessary to salvage the ischemic region from stroke. Unfortunately, reperfusion itself is also a major contributor to the final tissue damage and cardiac apoptosis. Searching for drugs that prevent cell death and cardiac tissue damage is a milestone for cardiovascular medicine. In addition, the protective potential of several molecules of diverse nature, such as RNA or DNA-related drugs. Further, the gain and loss of function of several lncRNAs have been proved both in vivo and in vitro. As previously described above, myocardial I/R injury increases ERS and UPR response and leads to increased cell apoptosis, caused by enhanced production of reactive oxygen species (ROS), impaired calcium handling and mitochondrial dysfunction [97]. Chen et al. (2019) described the downregulation of lncRNA UCA1 in the I/R injury model of H9C2 cardiomyocytes. Loss of function assays demonstrated that repression of UCA1 results in the upregulation of pivotal factors involved in UPR signalling, such as GRP78, ATF6 and PERK but not IRE1α. Enhanced expression of these factors reduced cell survival and increased intracellular levels of ROS. To address the possible role of UCA1 as a protective molecule against apoptosis, gain-of-function assays were performed showing that upregulation of UCA1 was capable of blocking ERS-associated apoptosis by repressing GRP78, ATF6 and PERK. In addition, overexpression of UCA1 reduces the production of ROS and improves mitochondrial function suggesting a potential role of this lncRNA as a possible protective factor in myocardial infarction and therefore heart failure. Regrettably, in vivo assays have not fully addressed yet the feasibility of UCA1 as a drug to improve cardiac recovery after HF [88] (Figure 3D and Figure 4).

Under I/R injury, endothelial cells respond by increasing the production of inflammatory factors such as cytokines and chemokines, which in turn enhances the migration of these cells and generates a proapoptotic environment. Similarly to UCA1, rcDLGAP4 displays reduced expression levels in the early phases of I/R recovery. Curiously, microRNA-143 exhibits an opposite expression pattern to circDLGAP4 during I/R injury displaying a peak expression in the advanced stages of injury. Functional assays showed that upregulation of circDLGAP4 results in decreased expression of ATF6 and migration of endothelial cells, but it does not modulate apoptosis signalling, suggesting that both processes are dependent on different molecular pathways. Mechanistically, circDLGAP4 exerts as a sponge of microRNA-143 impeding its binding to HECTD1, a pivotal ligase involved in the modulation of ERS on endothelial cells. HECTD1 reduces protein levels of ATF6 and its associated proapoptotic pathways. Furthermore, HECTD1 represses the migration of endothelial cells. Thus, the circDLGAP4-microRNA 143 complex increases the translation of HECTD1 protein which in turn leads to reduced ERS by blocking the ATF6 branch [89] (Figure 3E and Figure 4).

### 4.3. Cardiac Hypertrophy and Heart Failure

Heart hypertrophy requires cardiomyocyte growth resulting in increased protein synthesis in a short time span, generating a loss of ER homeostasis [98]. One of the core marks of heart hypertrophy and heart failure is metabolic derangement, which affects different genes involved in metabolic hypertrophy response such as 5′-adenosine monophosphate-activated protein kinase (*AMPK*), NAD-dependent deacetylase sirtuin-1 (*Sirt1*), NAD-dependent deacetylase (*NADD*) or Liver kinase B1 (*LKB1*). Zhu et al. (2022) identified low plasma levels of NRB2 lncRNA in patients with left ventricular hypertrophy. Induced hypertrophy by Angiotensin II (Ang II) administration in human cardiomyocytes revealed the downregulation of NRB2 as well as an increased level of cardiac hypertrophy markers such as ANF or BMP10. Upregulation of NRB2 on cardiac human cell lines results in severely reduced expression of cardiac hypertrophy markers and downregulation of ERS markers such as PERK, IRE1, GRP78 and CHOP. Moreover, the upregulation of NRB2 increases the expression of *LKB1*, *AMPK* and *Sirt1* suggesting that NRB2 may enhance the activation of the LKB1/AMPK/Sirt1 pathway. A loss of function assay of LKB1 was performed demonstrating that downregulation of LKB1 resulted in the weakened protective role of NBR2 on cardiac hypertrophy and ER stress. Taken together, NRB2 reduces myocardial hypertrophy by activating the LKB1/AMPK/Sirt1 pathway [90] (Figure 3F and Figure 4).

### 4.4. Dilated Cardiomyopathy

Dilated cardiomyopathy (DCM) is one of the main causes of heart failure exhibiting a prevalence of 7–10 million cases per year and approximately a 20% mortality rate. This cardiomyopathy is characterised by a progressive increase in ventricular size and contraction dysfunction of the left or both ventricles, without coronary artery diseases or changes in the pressure or load volume [99]. This affection appears in the third or fourth decade of life, displaying an incidence of 3:1 between man and woman suggesting a major prevalence in males as observed in other cardiovascular pathologies [100,101]. DCM hearts display an enhanced rate of cardiomyocyte apoptosis and intensive remodelling of the left ventricle which in turn can lead to heart failure. Several reports have pinpointed the crucial role of ERS in cardiomyocyte apoptosis associated with DCM. For example, Hamada et al. (2004) demonstrated that mutation of the Lys-Asp-Glu-Leu (KDEL) receptor in vivo results in the aggregation of misfolded proteins and increased cardiomyocyte apoptosis in mutant hearts by upregulation of CHOP. Curiously, transcriptional activation of CHOP is not exclusive to ATF6, IRE1 and PERK-related pathways [102]. Recently, Al-Yacoub et al. (2021) reported that a mutation in the *FBXO32* gene causes dilated cardiomyopathy by non-canonical activation of CHOP. Furthermore, heart explants from DCM patients exhibit increased expression of different ERS markers such as *ATF6*, *GRP78* or *XBP1* compared to controls demonstrating the activation of ERS and UPR pathways in DCM patients [30]. 

Transcriptome analysis of DCM hearts showed an intensive downregulation of AC061961.2, an unknown annotated lncRNA. Expression and functional analysis demonstrated that in vitro and in vivo Adriamycin-induced DCM reduces the expression of AC061961.2 while it enhances ERS-associated apoptosis by upregulating GRP78, CHOP, caspase 3 and Bax. Furthermore, Adriamycin treatment reduces protein levels of β-catenin, Axin-2 and c-Myc suggesting a downregulation of Wnt/β-catenin signalling. Gain-of-function assays of AC061961.2 reverses Adriamycin-induced apoptosis by activating Wnt/β-catenin signalling, increasing Bcl-2 expression and repressing protein levels of GRP78, CHOP and caspase 3 suggesting thus a role of AC061961 as a potential therapeutic drug against maladaptive UPR response [91] (Figure 3G and Figure 4).

### 4.5. Diabetic Cardiomyopathy

Diabetic cardiomyopathy is characterised by myocardial fibrosis, ER stress induction and cardiomyocyte cell death with the consequent cardiac dysfunction. Recently, Wang et al. (2022) have described a protector role of H19 lncRNA in the progression of this sickness by repression of ER stress. In a gain-of-function assay with H19 lncRNA in mice, these authors showed a reduction in cardiac dysfunction and cardiac chamber dilatation related to minor deposition of interstitial collagen and fibrosis. These effects were explained by the effect of H19 lncRNA in the ERS and the subsequent apoptosis. H19 induces the downregulation of ERS markers, such as ATF6, PERK, CHOP and IRE 1. These markers were upregulated in high glucose context and their presence induced UPR response and modified ER function. If the pathological stimulus persists, cardiac cells trigger an apoptotic program. The H19 effects previously detailed partially prevented cell death and the consequent alterations in heart function. Additionally, H19 alleviates ROS levels and indirectly reduces ROS-induced ERS [92]. Therefore, H19 exerts a key role in the progression of diabetic cardiomyopathy by the reduction of ERS and thus in the subsequent derived apoptosis (Figure 3H and Figure 4).

### 4.6. Limitations and Unresolved Issues

Although the studies discussed above demonstrate a pivotal role of both lncRNAs and circRNAs in the modulation of ERS and UPR pathways and in the progression of the associated CVDs, several limitations and unresolved issues should be pointed out. Firstly, functional assays have been performed in vitro which urges the implementation of the in vivo assays before confirming the therapeutic potential of these molecules in the CVD context. Secondly, only functions of NRB2 and AC061961 have been evaluated in human cardiomyocytes. This said, the role of the rest of the ncRNAs described above should be examined in human cardiac models first in vitro in order to evaluate the functional conservation between species and identify similar action on ERS and UPR modulation. Third, more exhaustive studies have to be conducted to glimpse the specific molecular mechanisms by which the described lncRNAs and circRNAs positively or negatively modulate the adaptive and maladaptive ERS responses. Furthermore, it would be interesting to determine the role of these ncRNAs in the progression of the adaptive response (protective role of UPR) towards the maladaptive response (harmful role of UPR). Taken together, the current knowledge establishes an illuminating starting point to better understand the role of the non-coding genome in ERS modulation and associated cardiovascular diseases.

## 5. Future Perspectives and Conclusions

Over the last few years, the impact of the non-coding genome has been widely proven in all cellular processes such as specification, differentiation, proliferation or homeostasis. The dysregulation of non-coding RNAs involved in several molecular pathways has been described in a multitude of diseases such as tumorigenesis, immune system disorders, or neurodegenerative and cardiovascular diseases. Likewise, the function of different non-coding RNAs has been related to the proper function and morphology of different cellular organelles, i.e., ER, cytoskeleton or mitochondria. Broadly, ER exerts a pivotal role in cellular proteostasis modulating the correct protein folding. Increased requirement of protein synthesis by different cellular or pathological injuries may result in disturbing proteostasis, increasing ERS and therefore triggering cellular homeostasis loss. To solve it, cells account for several protective complex molecular mechanisms such as UPR, ERAD and reticulophagy, which restore cellular proteostasis.

Several reports have highlighted ERS as both cause and consequence of distinct cardiovascular pathologies such as myocardial infarction, dilated cardiomyopathy or atherosclerosis. The regulatory potential of non-coding RNAs in ERS pathways has just begun to be explored, demonstrating both a protective–repressing UPRs activators such as ATF6, IRE1 or PERK or downstream genes related to apoptosis such as *CHOP*—and a harmful role—increasing maladaptive UPR and associated gene function. Gain-of-function and/or loss of function of the lncRNAs and circRNAs described above result in dramatic activation or repression of ERS, and thus apoptosis, suggesting their potential role as therapeutic targets and pinpointing to the complex molecular regulation of ERS. Nevertheless, most studies depicting the role of these RNAs have been performed in vitro while in vivo approaches are still limited. Furthermore, it would be necessary to delve into the upstream signal pathways regulating the transcriptional process of these lncRNAs and circRNAs related to ERS in order to achieve a better knowledge about the molecular environment orchestrating ERS response in distinct cardiovascular diseases.

## Figures and Tables

**Table 1:** Overview of lncRNAs and circRNAs involved in ERS-associated CVDs.

ncRNA	CVD	Effects	Mechanisms	Subjects and Size	Study Model	Type of the Study	Ref.
HypERlnc	Atherosclerosis	Inhibition of maladaptive UPR	Decrease in mRNA/protein levels of ATF6, IRE1α and Bip	Human cardiac tissue from patients with heart failure (HF) and pericytes exposed to hypoxia	Human	Gain and loss function assay ex vivo and in vitro	[85]
MEG3	Myocardial infarction	Activation of ERS-mediated apoptosis	Upregulation of mRNA/protein levels of GRP78, ATF4, PERK, eiF2α, CHOP and caspase 3	Infarcted hearts and hypoxic neonatal mice ventricular myocytes	Mice	Lost function assay in vitro	[86]
DANCR	Myocardial infarction	Inhibition of ERS-mediated apoptosis	Repression of GRP78, Beclin 1, p-IRE1α, p-IRE1α/IRE1α and Xbp1s by sponging miR-6324	H9C2 cardiomyocytes	Rat	Gain and loss function assay in vitro	[87]
UCA1	Ischaemia and reperfusion injury	Reduction of ROS production and improvement of mitochondrial function	Decrease in GRP78, ATF6 and PERK transcription	H9C2 cardiomyocytes	Rat	Gain and loss function assay in vitro	[88]
circDLGAP4	Ischaemia and reperfusion injury	Repression of ATF6 signalling pathway	Sponge to miR-143 avoiding to repression of HECTD1	Endothelial cells	Mouse	Gain function assay in vitro	[89]
NRB2	Heart hypertrophy and heart failure	Activation of LKB1/AMPK/Sirt1 pathway.	Decrease in mRNA/protein levels of PERK, IRE1, GRP78 and CHOP	Human cardiomyocytes cell line	Human	Gain-of-function assay in vitro	[90]
AC061961.2	Dilated cardiomyopathy	Reversion of apoptosis by activating Wnt/β-catenin signalling	Decrease in mRNA/protein levels of GRP78, CHOP and caspase 3	Vitro and in vivo Adriamycin-induced DCM	Rat	Gain-of-function assay in vitro	[91]
H19	Diabetic cardiomyopathy	Repression of cardiomyocyte apoptosis	Decrease in mRNA/protein levels of ATF6, PERK, CHOP and IRE1α	Induced DM mice	Mice	Gain function assay in vitro	[92]

## Data Availability

No new data were created or analyzed in this study. Data sharing is not applicable to this article.
